# Supplementary figures and images for: The Ets protein Pointed P1 represses Asense expression in type II neuroblasts by activating Tailless
Source: PLoS Genet. 2022 Jan 31;18(1):e1009928. doi: 10.1371/journal.pgen.1009928 (PMC8830786; doi:10.1371/journal.pgen.1009928)

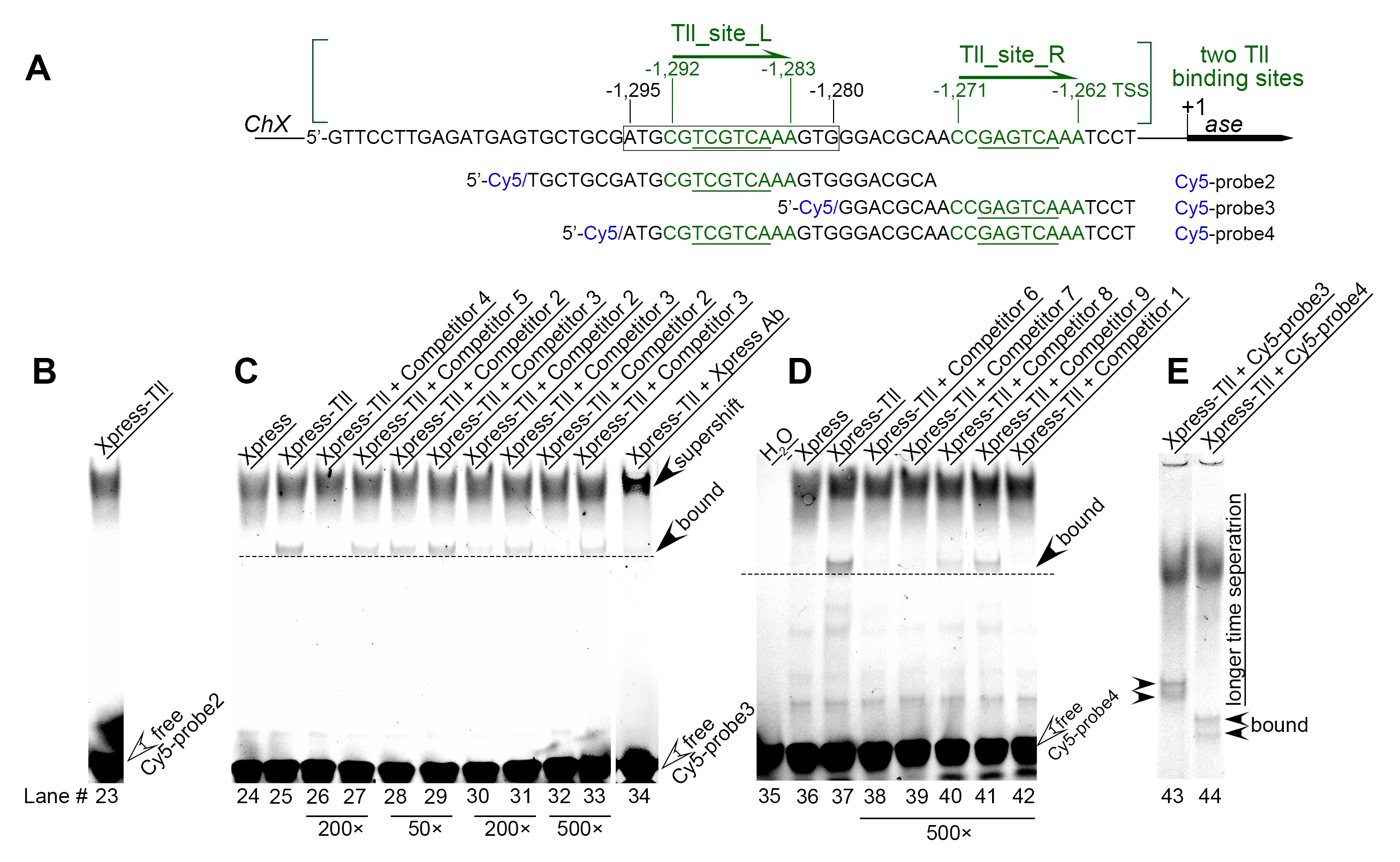

Supplement: S1 Fig — Open arrowheads point to free Cy5-probes at the bottom of gels, while filled arrowheads point to the lagged bands of the complexes of Tll-probes or super-shifted bands of the complexes of Xpress Ab-Tll-probes. Numbers below the lane numbers indicate the ratio of the competitor to the probe. The indicated competitors are the same as those shown in Fig 3. The lanes are numbered following the lane numbers in Fig 3. (A) The sequences of probes Cy5-probe2, Cy5-probe3, and Cy5-probe4 containing Tll_site_L, Tll_site_R, or both are shown. (B) No obvious binding is detected between Xpress-Tll and Cy5-Probe2 (lane #23), which contains Tll_site_L, possibly because the band is below the detection threshold. (C) The binding between Xpress-Tll and Cy5-probe3 and its competition by indicated competitors. Probe3 contains the sequence of Tll_site_R. Specific binding between Tll and Cy5-probe3 is detected in lane #25 and verified in lane #34 by the presence of a super-shifted band when the Xpress antibody is present. Both competitor 4, which has the same sequence as Cy5-probe3, and competitor 2, which contains the sequence of Tll_site_L, can compete with Cy5-probe3 for the binding with Xpress-Tll in a dose-dependent manner (lanes #26, #28, #30, and #32), but competitors (competitors 5 and 3) that contains only a mutated Tll_site_R or a mutated Tll_site_L cannot (lanes #27, #29, #31, and #33). (D) The binding between Xpress-Tll and Cy5-probe4 and its competition by indicated competitors. Probe4 contains both Tll_site_L and Tll_site_R. Cy5-probe4 binds to Xpress-Tll (lane #37). Both competitor 6 (lane #38), which contains the same sequence as Cy5-probe4, and competitor 1 (lane #42), which contains a Tll binding site from the kr enhancer, strongly competes with Cy5-probe4 for the binding with Xpress-Tll. Competitor 7 that contains a mutated Tll_site_L and the wild type Tll_site_R (lane #39) also strongly competes with Cy5-probe4 for binding with Xpress-Tll proteins. Competitor 8 that co [file pgen.1009928.s001.tif]

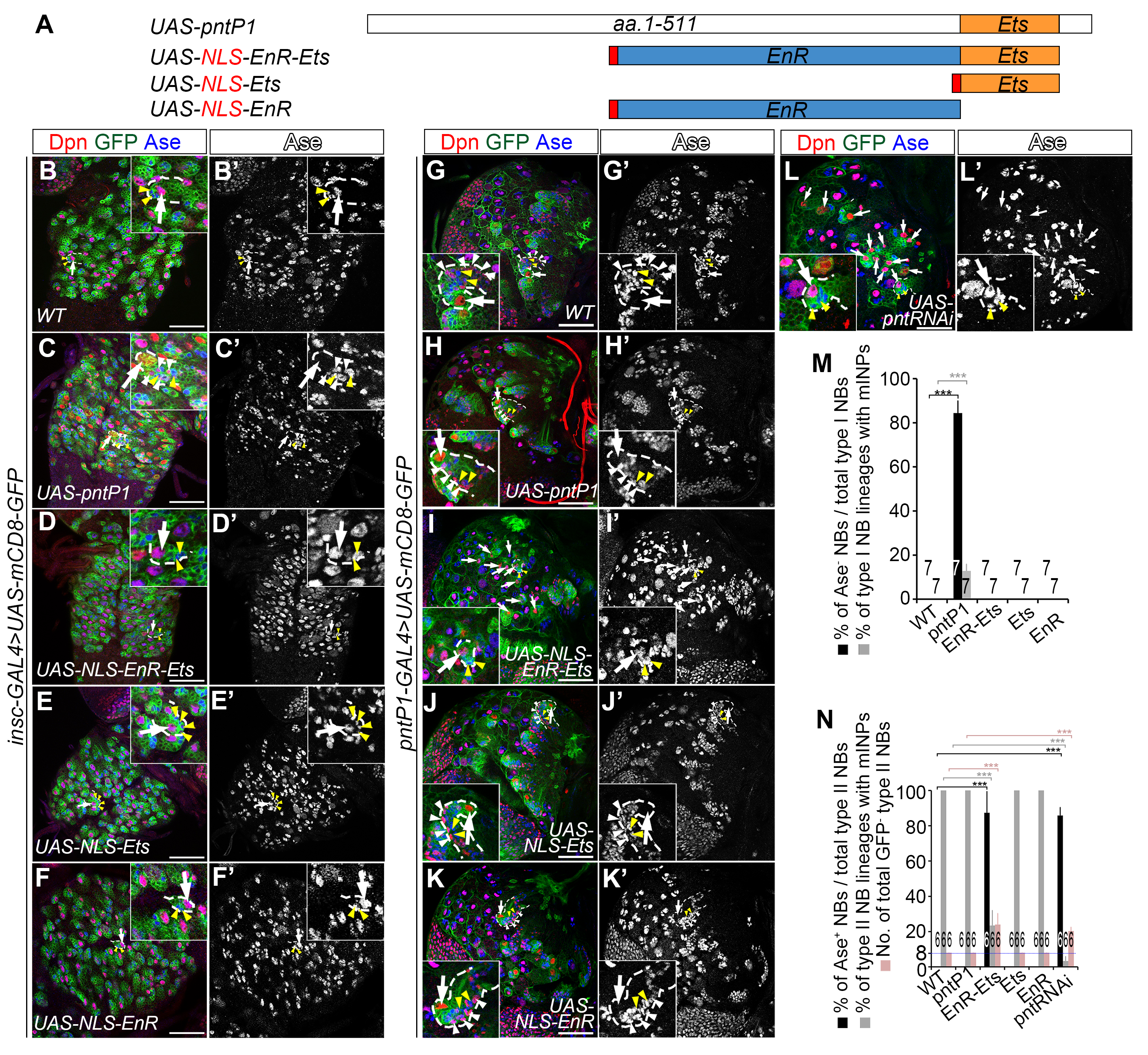

Supplement: S2 Fig — NB lineages are labeled with mCD8-GFP (in green) driven by insc-GAL4 for type I NB lineages (B-F) or by pntP1-GAL4 for type II NB lineages (G-L), and counterstained with anti-Dpn (in red) or anti-Ase (in blue) antibodies. White arrows point to some Ase+ Dpn+ type I NBs or Ase- Dpn+ type II NBs as examples. White arrowheads point to Ase+ Dpn+ mINPs or mINP-like cells. Yellow arrowheads point to Ase+ Dpn- GMCs. Dashed lines highlight representative NB lineages enlarged in insets. In this and all the following figures, only images from one brain lobe or the thoracic segments of VNCs are shown. The brain lobes are oriented so that the midline is to the right and the anterior side of the brain up. Scale bars equal 50μm. (A) Schematic diagrams of constructs used to express PntP1, NLS-EnR-Ets, NLS-Ets, or NLS-EnR. (B-B’) VNCs contain only Ase+ Dpn+ type I NBs, which directly produce Ase+ Dpn- GMCs. (C-C’) Misexpressing UAS-pntP1 in type I NBs represses Ase expression and induces Ase+ Dpn+ mINP-like cells. (D-F’) Misexpressing UAS-NLS-EnR-Ets (D-D’), UAS-NLS-Ets (E-E’) or UAS-NLS-EnR (F-F’) in type I NBs does not repress Ase expression or induce any mINP-like cells. (G-H’) A wild type (G-G’) or UAS-pntP1 overexpressing brain lobe (H-H’) has eight type II NB lineages, each of which contains an Ase- Dpn+ type II NB and multiple Ase+ Dpn+ mINPs. (I-I’) Type II NBs with UAS-NLS-EnR-Ets misexpression ectopically expresses Ase and directly produces Ase+ Dpn- GMCs instead of INPs. Note that UAS-NLS-EnR-Ets misexpression also leads to the generation of supernumerary type II NB (see white arrows) lineages. (J-K’) Misexpressing UAS-NLS-Ets (J-J’) or UAS-NLS-EnR (K-K’) does not affect the normal development of type II NB lineages, including the repression of Ase, the number of type II NBs, or the composition of cell types. (L-L’) Knocking down PntP1 by expressing UAS-pnt RNAi results in ectopic Ase expression in type II NBs, loss of INPs and generation of supernumerary type II NBs. (M [file pgen.1009928.s002.tif]

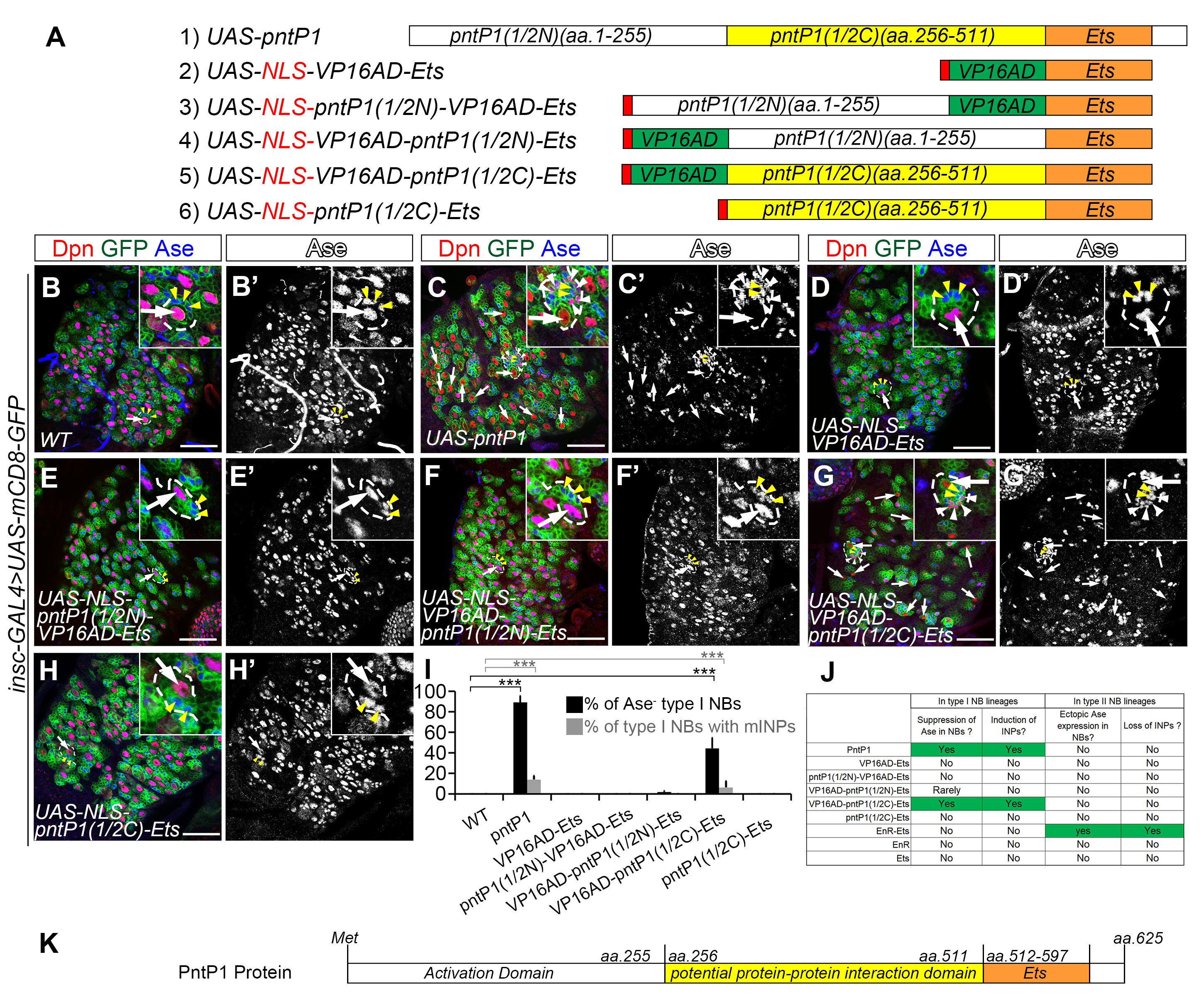

Supplement: S3 Fig — In all images, type I NB lineages in VNCs are labeled with mCD8-GFP (in green) driven by insc-GAL4, and counterstained with anti-Dpn (in red) or anti-Ase (in blue) antibodies. White arrows point to representative type I NBs in which Ase is expressed in (B-B’, D-D’, E-F’, and H-H’) or those in which Ase is repressed in (C-C’ and G-G’). White arrowheads point to induced Ase+ Dpn+ mINP-like cells and yellow arrowheads point to Ase+ Dpn- GMCs. Dashed lines highlight representative NB lineages enlarged in insets. Scale bars equal 50μm. (A) Schematic diagrams of constructs used to express PntP1, or sub-fragments of PntP1 with or without the fused VP16AD domain. (B-B’) VNCs contain only Ase+ Dpn+ type I NBs, which directly produce Ase+ Dpn- GMCs. (C-C’) Misexpressing UAS-pntP1 represses Ase expression in type I NBs and induces Ase+ Dpn+ mINP-like cells. (D-F’) Misexpressing chimeric activators NLS-VP16AD-Ets (D-D’), NLS-pntP1(1/2N)-VP16AD-Ets (E-E’) or NLS-VP16AD-pntP1(1/2N)-Ets (F-F’) does not suppress Ase expression in type I NBs. (G-H’) Misexpressing the chimeric activator NLS-VP16AD-pntP1(1/2C)-Ets represses Ase expression and induces Ase+ Dpn+ mINP-like cells (G-G’), while misexpressing NLS-pntP1(1/2C)-Ets does not (H-H’). (I) Quantifications of the percentage of type I NBs with Ase being suppressed or with mINP-like cells in VNCs expressing indicated chimeric proteins or wild type PntP1. Values of the bars are mean ± SD. ***, P < 0.001. (J) Summary of phenotypes resulting from the expression of PntP1, or indicated chimeric proteins in type I NB or type II NB lineages. (K) A diagram of functional domains of the PntP1 protein based on phenotypic analyses of the chimeric activator proteins. The potential activation domain is likely localized in the aa.1-255 region of PntP1, while the region of aa.256-511 is probably necessary for PntP1’s activity, potentially involved in recruiting co-factors, and the Ets domain (aa.512-597) is required for binding to target DNAs. (TIF) [file pgen.1009928.s003.tif]

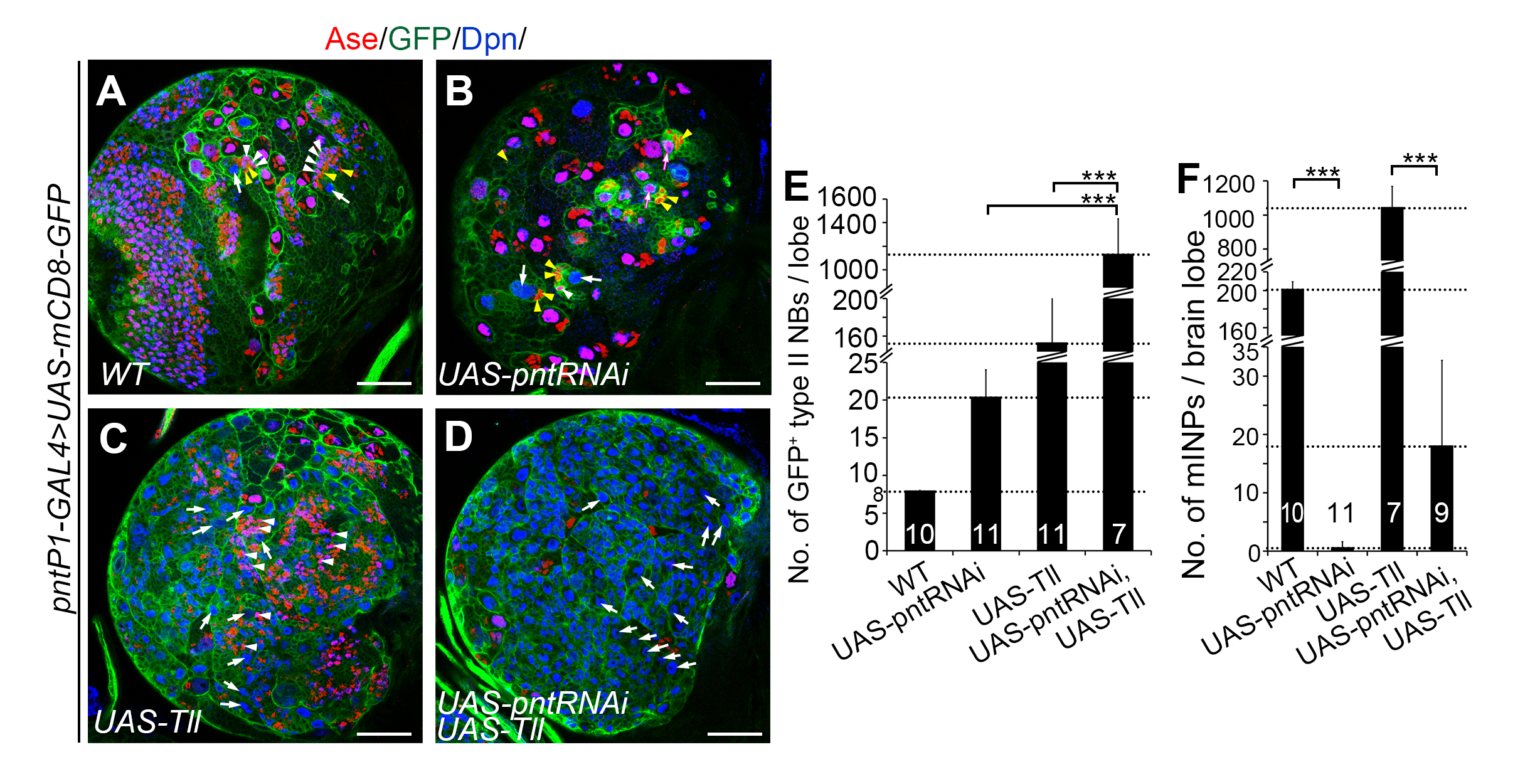

Supplement: S4 Fig — In all images, type II NB lineages are labeled with mCD8-GFP (in green) driven by pntP1-GAL4 and counterstained for Ase (in red) and Dpn (in blue). Arrows point to type II NBs, white arrowheads point to Ase+ Dpn+ mINPs, and yellow arrowheads point to Ase+ Dpn- GMCs. Scale bars equal 50μm. (A) A wild type brain lobe contains eight type II NBs, which are associated with their mINPs and GMCs. (B) Knockdown of PntP1 results in ectopic Ase expression in type II NBs (purple arrows filled with white), which directly produces GMCs at the expense of mINPs, and a slight increase in the total number of GFP labeled type II NBs. mINPs are also reduced or depleted in the lineages without the ectopic Ase expression in the NBs (e.g., white arrows). (C) Overexpressing Tll alone promotes the generation of supernumerary type II NBs (white arrows) but a large number of mINPs are still generated. (D) Simultaneous knockdown of PntP1 and overexpression of Tll leads to a dramatic increase in the number of type II NBs and near complete depletion of mINPs. (E-F) Quantifications of the number of type II NBs per lobe (E) or the total number of mINPs per lobe (F) in the brains with indicated genotypes. Values of the bars are mean ± SD. The numbers on each bar represent the number of brain lobes examined. ***, P < 0.001. (TIF) [file pgen.1009928.s004.tif]

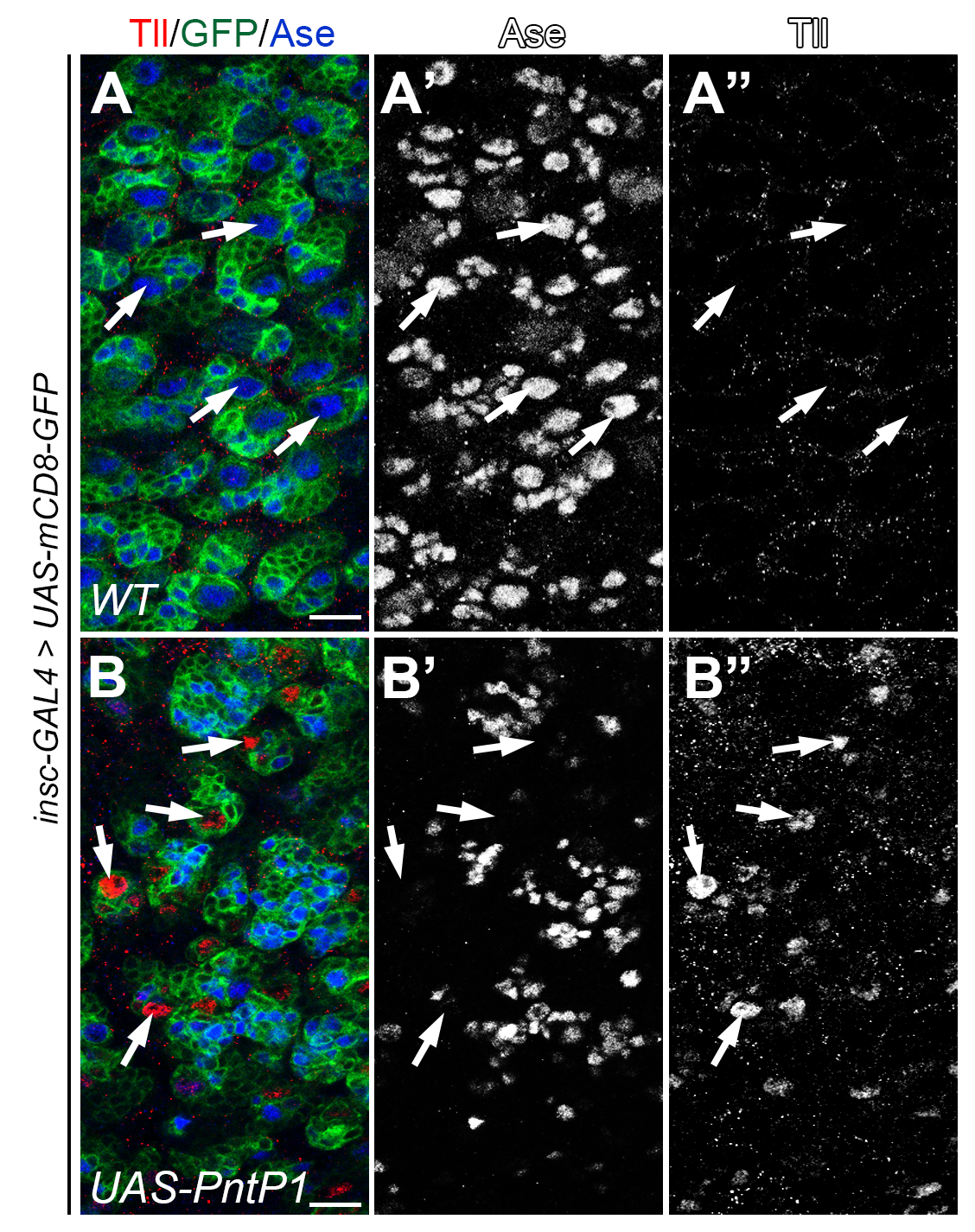

Supplement: S5 Fig — Type I NB lineages in the VNC are labeled with mCD8-GFP (in green) driven by insc-GAL4. The VNCs are counterstained with anti-Tll (in red), anti-Ase (in blue) antibodies. White arrows point to some representative type I NBs. Scale bars equal 10μm. (A-A”) Tll expression is not detected in type I NBs in VNCs. (B-B”) Misexpressing UAS-PntP1 activates ectopic Tll expression in all type I NBs and represses Ase expression. (TIF) [file pgen.1009928.s005.tif]

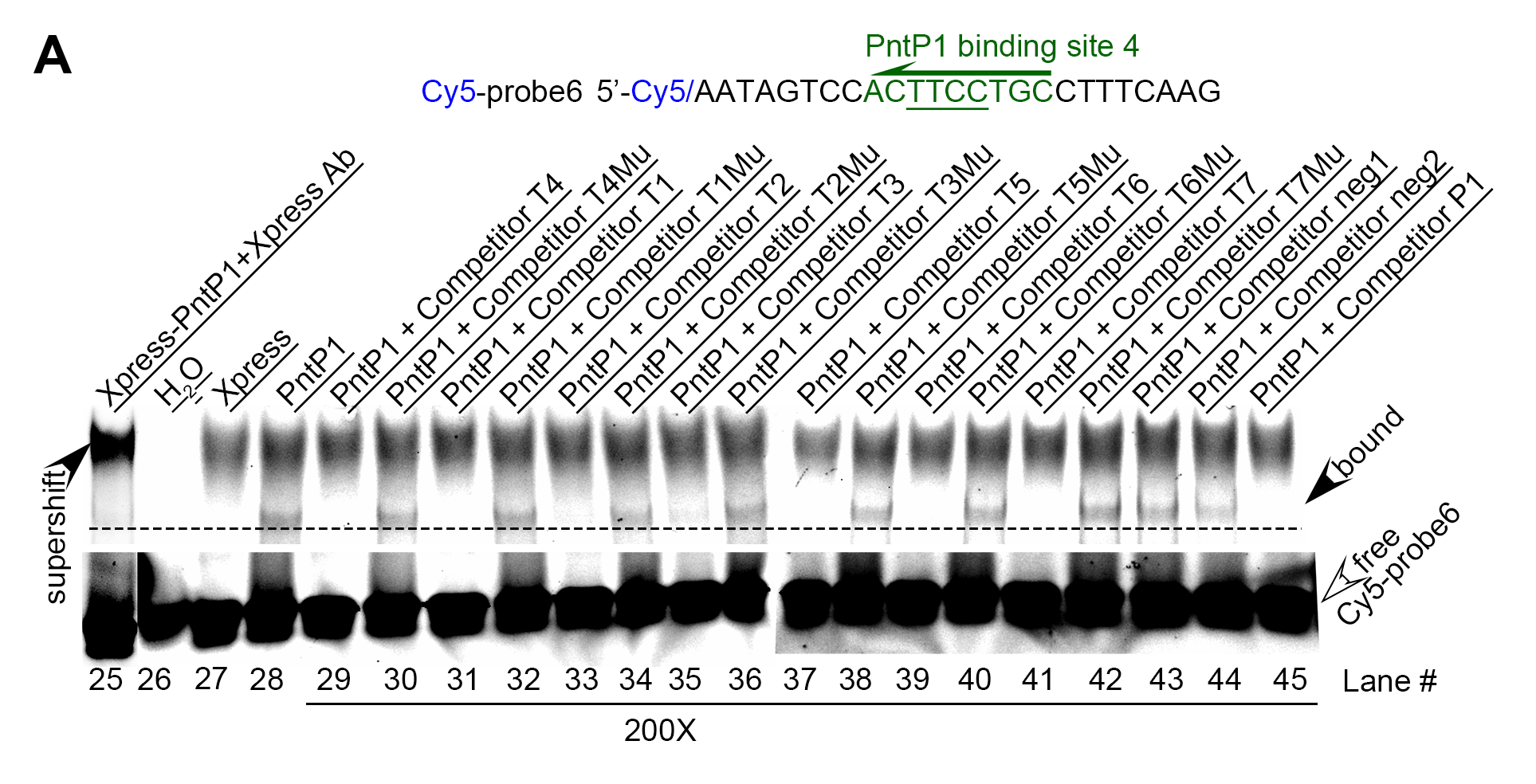

Supplement: S6 Fig — (A) Top: the sequence of Cy5-probe6 that contains the putative PntP1 binding site #4 shown in green. The green arrow indicates the orientation of the consensus PntP1-binding 5’-GGAA/T-3’ core sequence. The underlined sequence in the probe indicates the GGAA core sequence of the PntP1 binding motif. Bottom: binding of Cy5-probe6 with Xpress-PntP1 and its competition by the indicated competitors. The sequences of the competitors are the same as shown in Fig 5. Lanes are numbered following the lane numbers in Fig 5. Xpress-PntP1 proteins bind to Cy5-probe6 (lane #28). The super-shifted band in lane #25 indicates a further retardation on the mobility of the complex of Xpress Ab-PntP1-Cy5-probe6, when the anti-Xpress Ab is present. Both competitor T4 (lane #29), which contains the same sequence as Cy5-probe6, and competitor P1 (lane #45) can competes with Cy5-probe6 for the binding with Xpress-PntP1, but competitor T4Mu, which contains mutated PntP1 binding site #4 (lane #30) cannot. Competitors T1, T2, T3, T5, T6, or T7, which contains PntP1 binding sites #1, 2, 3, 5, 6, or 7 (corresponding to lanes #31, #33, #35, #37, #39, or #41), respectively, can competes with Cy5-probe6 for the binding to PntP1 to various extent, while competitors T1Mu, T2Mu, T3Mu, T5Mu, T6Mu, or T7Mu, which contains mutated binding site #1, 2, 3, 5, 6, or 7 (corresponding to lanes #32, #34, #36, #38, #40, or #42) or non-specific competitors neg#1 (lane #43) or neg#2 (lane #44), cannot. Open arrowheads point to free probes while filled arrowheads point to bands of the PntP1-probe complexes or the super-shifted bands of the Xpress Ab-PntP1-probe complexes. Numbers below the lane numbers indicate the ratio of the competitor to the probe. (TIF) [file pgen.1009928.s006.tif]

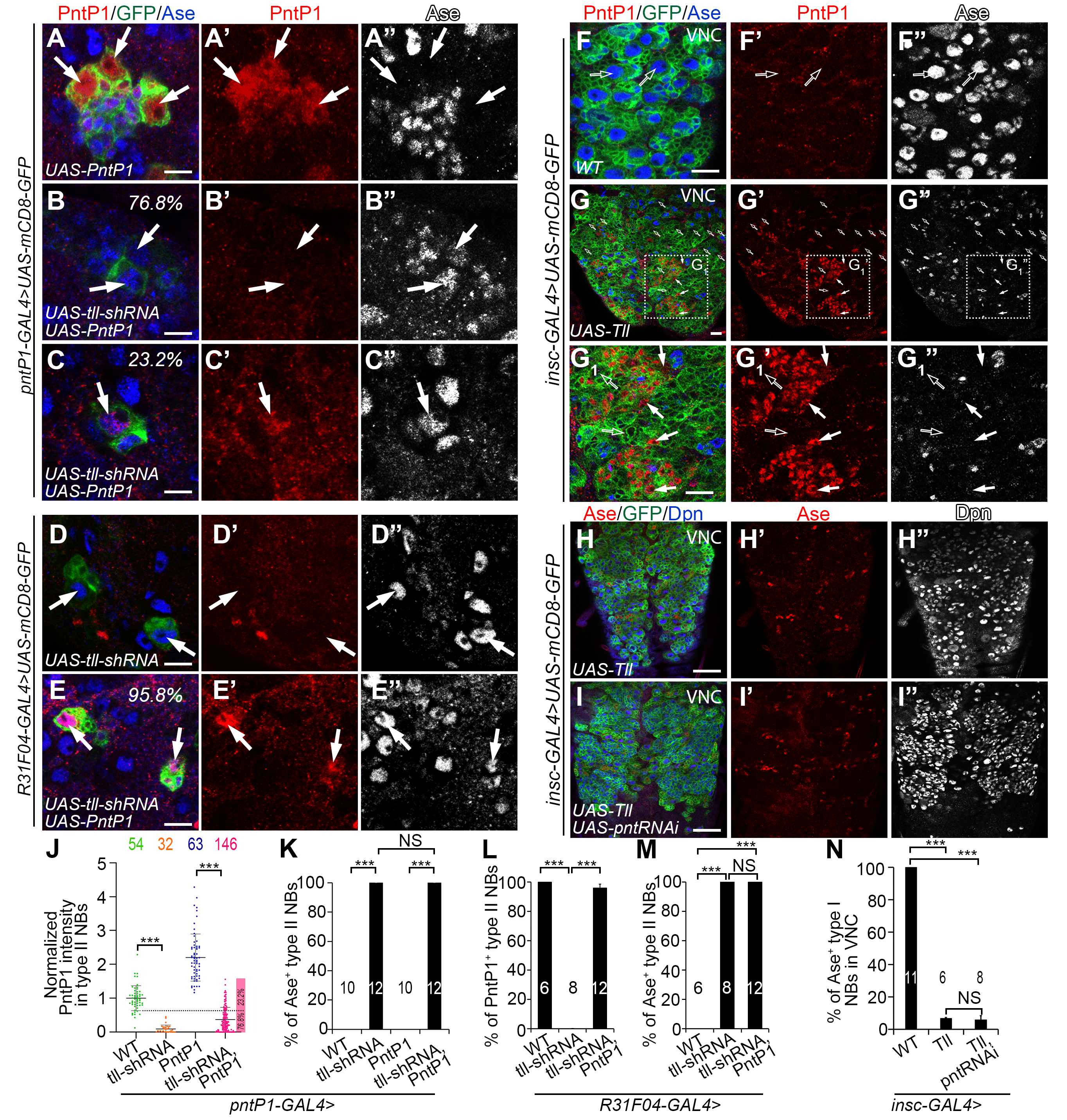

Supplement: S7 Fig — Type II NB lineages are labeled with mCD8-GFP (in green) driven by pntP1-GAL4 or R31F04-GAL4 and Type I NBs are labeled with mCD8-GFP (in green) driven by insc-GAL4. The brains and VNCs are counterstained with anti-Ase, anti-Dpn, and/or anti-PntP1 antibodies. White arrows point to type II NBs in (A-E”) or type I NBs with ectopic PntP1 expression in (F-G1”). Open arrows in (F-G1”) point to type I NBs without the ectopic PntP1 expression. Scale bars equal 10μm in A-G or 50μm in H-I. (A-C”) Expression of UAS-PntP1 driven by pntP1-GAL4 increases PntP1 levels in wild type type II NBs lineages (A-A’), and restores PntP1 expression in 23.2% of Tll knockdown type II NB lineages to levels comparable to those in wild type type II NB lineages (C-C”) but not in other 76.8% of Tll knockdown type II NB lineages (B-B”). However, Ase remains ectopically expressed in Tll knockdown type II NBs that have restored PntP1 levels (C-C”). (D-E”) Expression of UAS-tll-shRNA driven by R31F04-GAL4 leads to the loss of PntP1 and ectopic Ase expression in type II NBs (D-D”). Expression of UAS-PntP1 driven by R31F04-GAL4 restores PntP1 expression in 95.8% of Tll knockdown type II NBs. However, Ase remains ectopically expressed in all Tll knockdown type II NBs (E-E”). (F-G1”) PntP1 is never expressed in wild type type I NBs in the VNC (F-F”) and expressing UAS-Tll induces PntP1 expression in a subset of type I NBs but a majority of Tll misexpressing type I NBs still do not express PntP1 (G-G”). (G1-G1”) show an enlarged view of the area highlighted with a dotted square in (G-G”), respectively. (H-I”) Expressing UAS-Tll suppresses Ase expression and promotes the generation of supernumerary type I NBs (H-H”), and simultaneous knockdown of PntP1 does not restore Ase expression or inhibit the generation of supernumerary type I NBs (I-I”). (J-K) Quantifications of PntP1 staining intensity in type II NBs (J) and the percentage of Ase+ type II NBs (K) in animals with indicated phenotypes. The PntP1 inte [file pgen.1009928.s007.tif]
