# Supplementary material for: The Ets protein Pointed P1 represses Asense expression in type II neuroblasts by activating Tailless
Source: PLoS Genet. 2022 Jan 31;18(1):e1009928. doi: 10.1371/journal.pgen.1009928 (PMC8830786; doi:10.1371/journal.pgen.1009928)
Supplement: Attachment — Submitted filename: point-to-point responses to reviewers comments.docx [file pgen.1009928.s009.docx]

Point-to-point responses to reviewers’ comments

Reviewer #1: Asymmetric cell division is a mechanism to generate cell diversity, while maintaining a progenitor cell. Neuronal stem cells utilize ACD to form neuronal subtypes either directly or through the formation of intermediate progenitor cells. The mechanisms determining which pathway is used are incompletely understood. Here, Chen et al., use Drosophila neural stem cells, neuroblasts hereafter, to shed light on a transcriptional cascade that is used to specify type I, or intermediate progenitor forming (type II) neuroblasts. The authors provide evidence that the Ets protein Pointed induces the transcription of the transcriptional repressor Tailless. Tailless represses Asense expression in type II neuroblasts, thereby creating a subset of neuroblasts that are molecularly and functionally distinct from type I neuroblasts.
This is an interesting and detailed study that will be of interest to the neural stem cell field. The conclusions are predominantly supported by the presented data but I have two major concerns that need to be addressed.

(1) In my opinion, the use of chimeric constructs in Figure 1 and 2 to define whether Pointed acts as a transcriptional repressor vs. activator is borderline convincing mostly because the constructs are highly artificial and are inconclusive if they provide negative results. Did the authors consider generating Pointed deletion constructs, expressed in a pointed mutant background to define repressor vs. activator domains of pointed? The ase enhancer deletion analysis on the other hand is straightforward and convincing. I suggest moving the data shown in figure 1 and 2 to the supplementary figures and start

the story with figure 3.

Response: We would like to thank the reviewer for the constructive comments and suggestions. We agree that data from the artificially chimeric activators/repressors are preliminary and do not tell the precise boundaries of the activation domain or the potentially protein-protein domain. Thus, as the reviewer suggested, we started the story with the original Fig 3 (mapping of the *cis*-elements mediating the suppression of Ase in type II NBs) and moved the original Figs 1 and 2 to a later section as new Figs S2 and S3. However, we do plan to systematically generate a series of deletion constructs of PntP1 to fine map not only its activation domain but also other functional domains, which we hope to include in our future publications.

(2) Similarly, I find the logic of the experiments in figure 4 hard to follow and the data inconclusive. Since these constructs are expressed in a wild type background, how can the authors exclude the possibility that endogenous Tailless modulates the expression of the reporters? It would be more convincing to monitor ase expression in tailless mutants or under conditions of tailless misexpression in combination with the generated deletion constructs shown in figure 3 to define the tailless responsive element genetically.

Reponses: Sorry for the confusion. We revised the related text to make it more logically clear. However, we do not think that endogenous Tll has any significant role in regulating the expression of these GAL4 reporter lines. First, we did not detect obvious endogenous Tll expression in type I NBs in the VNC by immunostaining (please see the new data added in Figs 2K-2M” and new S5A-S5A”). Second, there was no obvious difference in the expression of *UAS-mCD8-GFP* driven by these GAL4 reporter lines when *UAS-tll* was not co-expressed (please see the new images in Fig 2G-2J, and 2Q). We wish we could make the data more convincing by repeating the same experiments in the *tll* mutant background or under conditions of Tailless misexpression in combination with the deletion constructs as the reviewer suggested, but it is genetically challenging to do these experiments, particularly within the 1-month time limit for the revision.

Minor critiques:

(3) Figure 2E, H, F, D lack high magnification inserts. These are necessary to verify the claims.

Response: we have added high magnification inserts into original Fig 2D-2F’ and 2H-2H’ (new S3D-S3F’ and S3H-S3H’ Fig in the revision).

Reviewer #2: The paper “PntP1 represses Asense by activating Tailless” aims to investigate the molecular mechanism underlying Ase suppression by PntP1 in type II NBs during the larval development. By constructing artificial proteins containing the Ets domain of PntP1 and a repressor/activator domain of another proteins, the authors suggested that instead of a direct transcriptional suppressor, PntP1 acts as an activator to indirectly repress ase expression. To identify the downstream effector of PntP1, a bottom-up approach was exploited in which the authors first explored the enhancer region of ase. By combining bioinformatics and molecular methods, the author mapped multiple sites in ase enhancer sequence required for the expression of Ase in type II or in all NBs. In a mapped sequence with repressive elements, a Tll binding motifs were identified in addition to another neighboring downstream of the repressive elements. Although EMSA results showed that both motifs in ase enhancer regions could bind to Tll in vitro, they appeared to have different affinity. Furthermore, PntP1 was demonstrated to be both necessary and sufficient for the expression of Tll. In addition, EMSA and ChIP-qPCR were utilized to show that PntP1 can directly bind to the enhancer region of tll in vitro and in vivo. On the other hand, Tll necessitates the maintenance of PntP1 expression. Interestingly, when PntP1 is expressed and Tll is knocked down in type I NBs, ectopic Ase expression remains. As such, it was suggested that PntP1 did not activate any other downstream effector in parallel to Tll to suppress Ase expression.
Overall: The methods used were appropriate to address the main research question although some details need further explanations. The manuscript provides excellent abstract and introduction that is clear and captured the essential knowledge necessary to understand further work. The provided data mostly supports the conclusions. However, some aspects of data visualization are a bit confusing. Additionally, statistical analysis this manuscript may require extra consideration.

Minor:
**- Methodology**

o EMSA: the first part in which the authors mentioned about this technique was a bit unclear. Therefore, it may need 1-2 sentence to explain how to read the data provided, for example, what to expect if the hypothesis is correct.

Response: we have added a couple of sentences to explain how EMSA works and how to read the data in lines #173-178.

o In the last section in which the authors investigated if PntP1 also regulates other downstream effectors to suppress Ase expression in parallel to Tll, pnt-Gal4>UAS-PntP1 in addition to Tll KD was used. In the results, UAS-PntP1 failed to restore PntP1 expression in this condition, likely due to the downregulation of pnt-Gal4 expression upon Tll KD due to the Tll-PntP1 positive feedback loop. Based on this and later in type I NBs with restored PntP1 expression, the authors concluded that Tll is the only target of PntP1 that controls Ase expression. Is there any type II NB-specific Gal4 that can drive UAS-PntP1 without being affected by Tll expression? If so, using this Gal4 alternatively of pnt-Gal4 may be able to strengthen the argument.

Response: We agree with the reviewer’s concern that the *pntP1-GAL4* driver is not perfect for this experiment. Therefore, as suggested by the reviewer, we also used *tll-GAL4* (i.e. *R31F04-GAL4*) to drive the expression of *UAS-PntP1*. This driver is supposedly not affected by the Tll expression (at least no studies show Tll regulates its own expression). Our new data added in the new S7D-S7E” and S7K-S7L Fig show that this driver can effectively restore PntP1 expression in nearly all type II NBs but Ase is still ectopically expressed in Tll knockdown type II NBs. These additional data further support our conclusion that Tll is the major downstream target of PntP1 that mediates the suppression of Ase.

o The authors only showed that Tll binds to ase enhancers in vitro via EMSA. However, in vivo evidence is lacking here. As Hakes and Brand (2020) previously performed TaDa to show that Tll binds to ase in vivo, the authors can either use another method (ChIP-qPCR) to validate or refer to this previous literature.

Response: we have added a sentence (lines #228-230) to cite a previous publication by Hakes and Brand (2020) that shows Tll binds to a 5 kb enhancer region of *ase* *in vivo*.

o ChIP-qPCR: Brat KD was used to enrich the type II NBs for ChIP-qPCR. Nevertheless, it was not discussed such as how using Brat KD may affect the results (e.g., effect on PntP1, Tll and their bindings)

Response: we have added a few sentences to discuss whether Brat KD would affect our ChIP-qPCR results in lines #461-471 of the revised manuscript.

**- Results and discussion**

o The paper of Hakes and Brand (2020) was mentioned in the section regarding Tll-PntP1 feedback loop, referring to when Tll is misexpressed, there is ectopic expression of PntP1. I did not fine this information in the original publication. Thus, the reference needs to be double-checked

Response: we double checked the paper of Hakes and Brand (2020) and did find the related information, which was shown in supplementary figures. In Figure 2-figure supplement 2C-C’, they showed that Pnt-GFP (a reporter of PntP1) was depleted in *tll* knockdown type II NBs. In Figure 4-figure supplement 1A’, they showed that misexpression of Tll leads to ectopic PntP1 expression in type I NBs.

o PntP1 was claimed to activate only Tll as a downstream effector to inhibit Ase expression. However, I think PntP1 can regulate many factors among which Tll is the key regulators. Others may still exit and regulate Ase expression, cooperatively with Tll. As such, the conclusion here may be a bit too far.

Response: we have made our conclusion less strong and revised the statement as follows “Tll is the major downstream target of PntP1 that is responsible for the suppression of Ase. Other downstream targets of PntP1 may not have significant roles in the suppression of Ase or just play a supplementary role by coordinating with Tll” (lines #408-411 of the revised manuscript).

o Limitations of the methods were not much discussed

Response: we have discussed limitations of some of the methods used in this study. For example, we have discussed whether using the Brat knockdown brains for ChIP-qPCR would give false results and whether we might miss some other PntP1 binding sites by limiting our bioinformatic prediction of PntP1 sites to the enhancer region *R31F04.*

**- Data representations**
o In figure 4: figure 4H was mentioned at the beginning of the section ‘Tll suppresses Ase expression directly by binding to ase enhancer). However, in the figure, it was placed after the others. Although it is understandable that the authors want to show the figures as a concluding model of the section, it causes confusion, the figures should be cited in chronological order to enhance readability.

Response: we have moved panels I and H to the beginning of this figure as panels A and B in the revised new Fig 2).

o Figure captions did not mention what data represents (e.g., mean ± SD)

Response: we have added Mean ± SD into the captions to indicate what quantification data represents.

o Figure 8H which showed the PntP1 intensity in type II NBs, if the data is normalized to the WT, it should be indicated in the axis label like other graphs in the manuscript (This is the only one that employed dot plot instead of bar graph).

Response: we have added the “normalized intensity” to the Y-axis of all the corresponding graphs, including the new Fig 2P and 2Q, the new Fig 4I and 4K, the new Fig 6 A”’, 6B”, and 6H (the original Fig 8H), and the new S7J Fig.

**- Statistical analysis**
o Student’s t-test was used throughout the manuscript regardless the sample number. As there are more than two samples in almost all cases of comparison, t-test may not be the most suitable. Accordingly, ANOVA needs to be used.

Response: Thanks to the reviewer’s suggestion. we have corrected the tests used in our statistical analyses. For the tests between two samples, t-tests (when the data are normally distributed) or U tests (when the data are not normally distributed) were used. For the tests among multiple samples, ANOVA were used first, and then multiple comparison were conducted by performing post-hoc tests, either Sidak test or Dunn's multiple comparison tests. All the statistical analyses done with these new methods gave consistent results as presented in the original manuscript. All the numeric data and their corresponding statistical analyses used were summarized and provided in the new supporting data in “S1 Data”.

**- Language and readability**

o There are some typos in the manuscript (e.g., anti-DesRed)

Response: Thanks to the reviewer for catching the typos. we have corrected all the typos.

o Some sentences are a bit too long and therefore difficult to read (e.g., The rational is that if Tll suppresses Ase…lack of suppression of Gal4 by Tll)

Response: we have split long sentences to make the manuscript easier to read.
